# Termination-of-resuscitation rule for emergency department physicians treating out-of-hospital cardiac arrest patients: an observational cohort study

**DOI:** 10.1186/cc13058

**Published:** 2013-10-13

**Authors:** Yoshikazu Goto, Tetsuo Maeda, Yumiko Nakatsu Goto

**Affiliations:** 1 Section of Emergency Medicine, Kanazawa University Hospital, 13-1 Takaramachi, Kanazawa 920-8641, Japan; 2 Department of Cardiology, Yawata Medical Center, 12-7 I Yawata, Komatsu 923-8551, Japan

## Abstract

**Introduction:**

The 2010 cardiopulmonary resuscitation guidelines recommend emergency medical services (EMS) personnel consider prehospital termination-of-resuscitation (TOR) rules for out-of-hospital cardiac arrest (OHCA) following basic life support and/or advanced life support efforts in the field. However, the rate of implementation of international TOR rules is still low. Here, we aimed to develop and validate a new TOR rule for emergency department physicians to replace the international TOR rules for EMS personnel in the field. This rule aims to guide physicians in deciding whether to withhold further resuscitation attempts or terminate on-going resuscitation immediately after patient arrival.

**Methods:**

We analyzed data prospectively collected in a nationwide Utstein-style Japanese database between 2005 and 2009, from 495,607 adult patients with OHCA. Patients were divided into development (*n* = 390,577) and validation (*n* = 105,030) groups. The main outcome measures were specificity, positive predictive value (PPV), and area under the receiver operating characteristic (ROC) curve for the newly developed TOR rule.

**Results:**

We developed a new TOR rule that includes 3 criteria based on the results of multivariate logistic regression analysis for predicting a 1-month death after OHCA: no prehospital return of spontaneous circulation (adjusted odds ratio [OR], 25.8; 95% confidence interval [CI], 24.7–26.9), unshockable initial rhythm (adjusted OR, 2.76; 95% CI, 2.54–3.01), and unwitnessed by bystanders (adjusted OR, 2.18; 95% CI, 2.09–2.28). The specificity, PPV, and area under the ROC curve for this new TOR rule for predicting 1-month death in the validation group were 0.903 (95% CI, 0.894–0.911), 0.993 (95% CI, 0.992–0.993), and 0.874 (95% CI, 0.872–0.876), respectively.

**Conclusions:**

We developed and validated a new TOR rule for emergency department physicians consisting of 3 prehospital variables (no prehospital ROSC, unshockable initial rhythm, and unwitnessed by bystanders) that is a >99% predictor of very poor outcome. However, the implementation of this new rule in other countries or EMS systems requires further validation studies.

## Introduction

Out-of-hospital cardiac arrest (OHCA) has a poor prognosis and is a leading cause of death in the developed world. The incidence of OHCA treated by emergency medical services (EMS) personnel has been estimated to be approximately 275,000 persons per year, with a survival rate of 10.7% for all initial rhythm in Europe [1] and approximately 300,000 persons per year with a survival rate of 9.6% in United States [2]. Despite decades of research, the survival rates after OHCA have remained virtually unchanged in the past three decades [3,4].

The 2010 American Heart Association (AHA) Guidelines for Cardiopulmonary Resuscitation and Emergency Cardiovascular Care [5] recommend that EMS personnel consider prehospital termination of resuscitation (TOR) for patients with OHCA following basic life support (BLS) and/or advanced life support (ALS) efforts in the field. The prehospital BLS TOR rule with three criteria––unwitnessed by EMS personnel, no shock given and no prehospital return of spontaneous circulation (ROSC)––for EMS personnel was originally developed in Toronto by Verbeek *et al*. [6] and has been validated worldwide [7-10]. The authors of the original BLS TOR rule derived an ALS TOR rule with two additional criteria [11]. These TOR rules for OHCA have been implemented to better utilize hospital healthcare resources, reduce the number of attendant hazards to EMS personnel and the considerable associated financial expense, and increase the availability of care and transport for other patients [6-12].

The decision to terminate resuscitation efforts is fraught with ethical and legal considerations [5]. Therefore, any guidelines for TOR in the field must be highly reliable, accurate and legally defensible [9]. Currently, the rate of implementation of the TOR rules is low [13-16]. Different TOR rules, other than the aforementioned BLS and ALS TOR rules, should be established with reliability in different EMS systems. EMS personnel in Japan, however, are not legally allowed to terminate resuscitation for OHCA patients in the field; therefore, almost all OHCA patients are transported to a hospital, regardless of whether resuscitation is successful. Any TOR rules in the prehospital settings are thus not legally implemented in Japan. Therefore, a new TOR rule for emergency department physicians is required to replace the international TOR rules for EMS personnel in the field to allow for better utilization of hospital healthcare resources.

In this study, we aimed to establish and validate a new TOR rule for emergency department physicians that would allow them to decide whether to withhold further resuscitation attempts or terminate ongoing resuscitation immediately after patient arrival. Moreover, we validated the BLS TOR rule to compare the relevance of a new TOR rule.

## Materials and methods

### Study design and data source

The present study was a nationwide population-based observational study in all adult patients ages 18 years and older for whom resuscitation had been attempted after OHCA in Japan from 1 January 2005 to 31 December 2009. *Cardiac arrest* was defined as the cessation of cardiac mechanical activities confirmed by the absence of signs of circulation [17]. The cause of arrest was presumed to be of cardiac origin unless evidence suggested external causes (trauma, hanging, drowning, drug overdose and asphyxia), respiratory diseases, cerebrovascular diseases, malignant tumors or any other noncardiac causes. Attribution of noncardiac or cardiac etiology was made by the physicians in charge in collaboration with the EMS personnel. This study was approved by the Ethical Committee of Kanazawa University. The requirement for written informed consent was waived.

### Emergency medical services system in Japan

Japan has approximately 127 million residents in an area of 378,000 km^2^, approximately two-thirds of which is uninhabited mountainous terrain [18]. Details on the Japanese EMS system have been described elsewhere [18,19]. Briefly, municipal governments provide EMS through approximately 800 fire stations with dispatch centers. The Fire and Disaster Management Agency (FDMA) of Japan supervises only the EMS system nationwide, and individual EMS systems are operated by each local fire station. Generally, an ambulance crew consists of three EMS staff members, including at least one emergency lifesaving technician (ELST). ELSTs are allowed to use several resuscitation methods, including semiautomated external defibrillators, insertion of an adjunct airway, insertion of a peripheral intravenous line and administration of Ringer lactate solution. Since July 2004, only specially trained ELSTs have been permitted to insert a tracheal tube, and since April 2006, they have been permitted to administer intravenous epinephrine in the field under online physician instruction. Since October 2006, all EMS providers have performed cardiopulmonary resuscitation (CPR) according to the Japanese CPR guidelines [17], which are based on the 2005 AHA Guidelines for Cardiopulmonary Resuscitation and Emergency Cardiovascular Care [20]. As EMS personnel in Japan are legally prohibited from terminating resuscitation in the field, most OHCA patients undergo CPR by EMS providers and are transported to hospitals, except in cases where fatality is certain [18]. The length of the on-scene effort by EMS personnel is not predetermined before transport is initiated. Epinephrine use is implemented according to the FDMA resuscitation guidelines for ELSTs [17,21]. The permitted dosage of epinephrine is 1 mg per attempt, and repeated doses may be administered under the physician’s instruction.

### Data collection and quality control

The FDMA launched a prospective population-based observational study of all OHCA victims who received EMS in Japan [18] since January 2005. EMS personnel at each center, using an Utstein-style template, recorded data for OHCA victims with the cooperation of the physician in charge [22]. All data were transferred and stored in a nationwide database developed by the FDMA for public use. For this study, we analyzed this anonymous database with the permission of the FDMA. The main items included in the database are patient sex, age, etiology of arrest (presumed cardiac origin or not), bystander witness status, bystander CPR with or without automated external defibrillator use, initial identified cardiac rhythm, bystander category (that is, if there was a bystander, then whether the bystander was a layperson or EMS personnel), whether epinephrine was administered, whether advanced airway management (including endotracheal tube, laryngeal mask airway and esophageal-tracheal tube) were used, whether ROSC was achieved before arrival at the hospital, time of the emergency call, time of vehicle arrival at the scene, time of ROSC, time of vehicle arrival at the hospital, one-month survival and neurological outcome at one month after cardiac arrest. *Neurological outcome* was defined on the basis of the Cerebral Performance Category (CPC) scale: category 1, good cerebral performance; category 2, moderate cerebral disability; category 3, severe cerebral disability; category 4, coma or vegetative state; and category 5, death [22]. This CPC categorization was determined by the physicians in charge. The call-to-response time was calculated as the time from the emergency call to the time of vehicle arrival at the scene. The call-to-hospital arrival time was calculated as the time from the emergency call to the time of vehicle arrival at the hospital.

### Main outcome measures

The main outcome measures were specificity, positive predictive value (PPV) and area under the receiver operating characteristic (ROC) curve for the newly developed TOR rule for emergency department physicians. Secondary outcome measures were those related to the BLS TOR rule.

### Statistical analysis

Kolmogorov–Smirnov and Lilliefors tests were performed to evaluate the distributions of continuous variables, and we found that all continuous variables were not normally distributed (all *P* < 0.01). Therefore, the Wilcoxon and Kruskal–Wallis tests for continuous variables and the χ^2^ test for categorical variables were performed to compare the basic characteristics of patients between the development and validation groups. Multivariate logistic regression analyses including 11 variables were performed to assess the association between prehospital variables and one-month death or unfavorable neurological outcome in the development group. Continuous variables are expressed as medians (interquartile range (IQR)) [1st to 3rd quartiles], and categorical variables are expressed as percentages. As an estimate of effect size and variability, we report odds ratios (ORs) with 95% confidence intervals (CIs). All statistical analyses were performed with the JMP version 10 statistical discovery software package (SAS Institute Inc, Cary, NC, USA). All tests were two-tailed, and *P* < 0.05 was considered statistically significant.

## Results

**Figure 1:**
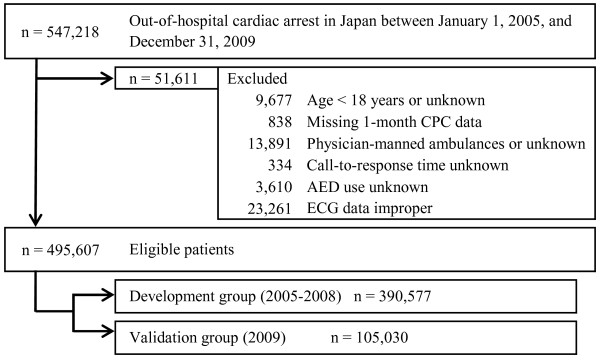
**Study profile and selection of patient population.** AED, automated external defibrillator; CPC, cerebral performance category; ECG, electrocardiogram.

During the five-year study period, 547,218 patients were documented in the database. From among these patients, 495,607 (90.6%) were eligible for enrollment in this study. Figure 1 shows a flow diagram depicting the inclusion and exclusion criteria for patients in the present study. Patients were divided into a development group (2005 to 2008; *n* = 390,577) and a validation group (2009; *n* = 105,030). The characteristics of all patients and the results of analyses of the two groups are shown in Table 1. Because of the large size of the study population, several significant differences were noted in baseline characteristics between the two groups; however, sizeable differences were less frequent, except for the ratio of bystander CPR. Overall rates of one-month survival and CPC categories 1 and 2 were 3.93% and 1.69%, respectively. The validation group had significantly higher one-month survival and one-month CPC categories 1 and 2 rates than the development group (survival: 4.34% vs. 3.81%; CPC categories 1 and 2: 2.04% vs. 1.60%; all *P* < 0.0001).

**Table 1:** **Baseline characteristics of the study patients**^**a**^

**Characteristics**	**All patients**	**Development group, 2005 to 2008**	**Validation group, 2009**	***P-*** **value**
	**(*N *** **= 495,607, 100%)**	**(*n *** **= 390,577, 78.8%)**	**(*n *** **= 105,030, 21.2%)**	
Age, years	76 (64 to 84)	76 (64 to 84)	77 (65 to 85)	<0.0001
Males	290,712 (58.7%)	229,822 (58.8%)	60,890 (58.0%)	<0.0001
Witnessed cardiac arrest	188,471 (38.0%)	147,997 (37.9%)	40,474 (38.5%)	<0.0001
Cardiac arrest witnessed by EMS personnel	22,722 (4.6%)	17,514 (4.5%)	5,208 (5.0%)	<0.0001
Bystander CPR	202,827 (40.9%)	151,712 (38.8%)	51,115 (48.7%)	<0.0001
Presumed cardiac etiology	276,183 (55.7%)	216,242 (55.4%)	59,941 (57.0%)	<0.0001
Shockable initial cardiac rhythm	38,388 (7.75%)	30,154 (7.7%)	8,234 (7.8%)	0.1992
Prehospital AED administration	53,427 (10.8%)	42,216 (10.8%)	11,211 (10.7%)	0.212
Call-to-response time, minutes	9 (7 to 11)	6 (5 to 9)	7 (5 to 9)	<0.0001
Call-to-hospital arrival time, minutes	29 (23 to 36)	29 (23 to 36)	30 (24 to 37)	<0.0001
Prehospital ROSC	25,254 (5.1%)	18,835 (4.8%)	6,419 (6.1%)	<0.0001
One-month outcome after cardiac arrest				
Survival	19,453 (3.93%)	14,898 (3.81%)	4,555 (4.34%)	<0.0001
Favorable neurological outcome (CPC category 1 or 2)	8,391 (1.69%)	6,244 (1.60%)	2,147 (2.04%)	<0.0001

^a^AED, automated external defibrillator; CPC, Cerebral Performance Category; CPR, cardiopulmonary resuscitation; EMS, emergency medical services; ROSC, return of spontaneous circulation. Values are reported as either number of patients (%) or medians (interquartile range [1st to 3rd quartiles]).

### Development of a new termination-of-resuscitation rule for emergency department physicians

The results of multivariate logistic regression analyses, including 11 variables to determine the factors associated with one-month death and CPC categories 3 to 5, are shown in Table 2. No prehospital ROSC had the highest adjusted OR for one-month death (adjusted OR, 25.8; 95% CI, 24.7 to 26.9) followed by unshockable initial rhythm (adjusted OR, 2.76; 95% CI, 2.54 to 3.01) and unwitnessed by bystanders (adjusted OR, 2.18; 95% CI, 2.09 to 2.28). We selected three prehospital variables that had adjusted ORs greater than 2.0 for predicting one-month death as predictors for one-month outcomes. Therefore, these three variables were incorporated into the new TOR rule, meaning that TOR was newly defined as appropriate in cases fulfilling all three criteria: no prehospital ROSC, unshockable initial rhythm and unwitnessed cardiac arrest by bystanders. No prehospital ROSC was also strongly associated with one-month CPC categories 3 to 5 with an adjusted OR of 38.4 (95% CI, 35.9 to 41.0).

**Table 2:** **Results of univariate and multivariate logistic regression analyses for factors associated with one-month outcomes**^**a**^

**Variables**	**Death**		**CPC categories 3 to 5**	
	**Crude OR (95% CI)**	**Adjusted OR (95% CI)**	**Crude OR (95% CI)**	**Adjusted OR (95% CI)**
Age^b^	1.02 (1.02 to 1.02)	1.01 (1.01 to 1.01)	1.03 (1.03 to 1.03)	1.03 (1.02 to 1.03)
Gender (male)	1.40 (1.35 to 1.45)	1.00 (0.96 to 1.05)	0.53 (0.50 to 0.56)	0.90 (0.84 to 0.96)
Unwitnessed by bystanders	4.70 (4.54 to 4.88)	2.18 (2.09 to 2.28)	7.04 (6.61 to 7.50)	2.01 (1.87 to 2.17)
Unwitnessed by EMS personnel	3.51 (3.33 to 3.68)	1.69 (1.59 to 1.81)	5.10 (4.77 to 5.44)	2.68 (2.46 to 2.93)
No bystander CPR	1.13 (1.10 to 1.17)	1.01 (0.97 to 1.06)	1.29 (1.23 to 1.36)	1.11 (1.04 to 1.18)
Noncardiac etiology	1.38 (1.34 to 1.43)	0.93 (0.89 to 0.97)	3.00 (2.83 to 3.19)	1.84 (1.71 to 1.99)
Unshockable initial rhythm	10.4 (10.0 to 10.8)	2.76 (2.54 to 3.01)	21.6 (20.5 to 22.7)	3.38 (2.98 to 3.84)
No prehospital AED administration	7.50 (7.25 to 7.76)	1.54 (1.41 to 1.67)	15.2 (14.4 to 16.0)	1.42 (1.24 to 1.61)
No prehospital ROSC	43.4 (41.8 to 45.0)	25.8 (24.7 to 26.9)	80.2 (75.5 to 85.2)	38.4 (35.9 to 41.0)
Call-to-response time^b^	1.10 (1.10 to 1.11)	1.05 (1.04 to 1.06)	1.13 (1.12 to 1.14)	1.05 (1.04 to 1.06)
Call-to-hospital arrival time^b^	1.02 (1.01 to 1.02)	1.03 (1.02 to 1.03)	1.02 (1.02 to 1.02)	1.02 (1.02 to 1.03)

^a^AED, automated external defibrillator; CI, confidence interval; CPC, cerebral performance category; CPR, cardiopulmonary resuscitation; EMS, emergency medical services; OR, odds ratio; ROSC, return of spontaneous circulation. ^b^Adjusted odds ratios are reported for unit odds.

### Analysis of derivation and validation datasets of the new termination-of-resuscitation rule

Table 3 shows the results of the analysis of derivation (development group) and validation (validation group) data sets of the new TOR rule for predicting one-month death. The rates of patients who fulfilled all three criteria in the development and validation groups were 57.1% and 57.3%, respectively, both of which would be considered futile. The new TOR rule showed a specificity of 0.875 (95% CI, 0.870 to 0.881), PPV of 0.992 (95% CI, 0.991 to 0.992) and area under the ROC curve of 0.851 (95% CI, 0.850 to 0.852) in the development group. The specificity, PPV and area under the ROC curve for the validation group were 0.903 (95% CI, 0.894 to 0.911), 0.993 (95% CI, 0.992 to 0.993) and 0.874 (95% CI, 0.872 to 0.876), respectively. Table 4 shows the results of the analysis of derivation and validation data sets of the new TOR rule for predicting a one-month unfavorable neurological outcome. The new TOR rule showed a specificity of 0.939 (95% CI, 0.933 to 0.945), PPV of 0.998 (95% CI, 0.998 to 0.999) and area under the ROC curve of 0.922 (95% CI, 0.921 to 0.923) in the development group. The specificity, PPV and area under the ROC curve for the validation group were 0.966 (95% CI, 0.958 to 0.973), 0.999 (95% CI, 0.999 to 0.999) and 0.942 (95% CI, 0.941 to 0.944), respectively.

**Table 3:** Performance of the new termination-of-resuscitation rule for predicting one-month death^**a**^

	**Development group (*N *** **= 390,577)**		**Validation group (*N *** **= 105,030)**	
**Variables**	**Fulfilled 3/3 criteria**	**Did not fulfill criteria**	**Fulfilled 3/3 criteria**	**Did not fulfill criteria**
	**(*n *** **= 223,023, 57.1%)**	**(*n *** **= 167,554, 42.9%)**	**(*n *** **= 60,205, 57.3%)**	**(*n *** **= 44,825, 42.7%)**
Death, *n*	221,167	154,512	59,763	40,712
Survival, *n*	1,856	13,042	442	4,113
Sensitivity (95% CI)	0.589 (0.587 to 0.590)		0.595 (0.592 to 0.598)	
Specificity (95% CI)	0.875 (0.870 to 0.881)		0.903 (0.894 to 0.911)	
PPV (95% CI)	0.992 (0.991 to 0.992)		0.993 (0.992 to 0.993)	
NPV (95% CI)	0.078 (0.077 to 0.079)		0.092 (0.089 to 0.095)	
Area under the ROC curve (95% CI)	0.851 (0.850 to 0.852)		0.874 (0.872 to 0.876)	

^a^CI, confidence interval; PPV, positive predictive value; NPV, negative predictive value; ROC, receiver operating characteristic.

**Table 4:** **Performance of the new termination-of-resuscitation rule for predicting one-month unfavorable neurological outcome**^**a**^

	**Development group (*N *** **= 390,577)**		**Validation group (*N *** **= 105,030)**	
**Variables**	**Fulfilled 3/3 criteria**	**Did not fulfill criteria**	**Fulfilled 3/3 criteria**	**Did not fulfill criteria**
	**(*n *** **= 223,023, 57.1%)**	**(*n *** **= 167,554, 42.9%)**	**(*n *** **= 60,205, 57.3%)**	**(*n *** **= 44,825, 42.7%)**
Unfavorable (CPC categories 3 to 5), *n*	222,642	161,691	60,132	42,751
Favorable (CPC categories 1 and 2), *n*	381	5,863	73	2,074
Sensitivity (95% CI)	0.579 (0.578 to 0.581)		0.585 (0.582 to 0.588)	
Specificity (95% CI)	0.939 (0.933 to 0.945)		0.966 (0.958 to 0.973)	
PPV (95% CI)	0.998 (0.998 to 0.999)		0.999 (0.999 to 0.999)	
NPV (95% CI)	0.035 (0.034 to 0.036)		0.046 (0.044 to 0.048)	
Area under the ROC curve (95% CI)	0.922 (0.921 to 0.923)		0.942 (0.941 to 0.944)	

^a^CPC, Cerebral Performance Category; CI, confidence interval; PPV, positive predictive value; NPV, negative predictive value; ROC, receiver operating characteristic.

### Analysis of validation dataset of the basic life support termination-of-resuscitation rule

Table 5 shows the results of analysis of the 2009 validation data set (*N* = 105,030) of the BLS TOR rule for predicting one-month death. The rate of patients who fulfilled all three criteria of the BLS rule in the validation group was 81.6%, which would be considered futile. The BLS TOR rule showed a specificity of 0.779 (95% CI, 0.767 to 0.791), PPV of 0.988 (95% CI, 0.988 to 0.989) and area under the ROC curve of 0.811 (95% CI, 0.809 to 0.813). Table 6 shows the results of the analysis of the validation data set of the BLS TOR for predicting one-month unfavorable neurological outcomes. The BLS TOR rule showed a specificity of 0.928 (95% CI, 0.917 to 0.938), PPV of 0.998 (95% CI, 0.997 to 0.998) and area under the ROC curve of 0.880 (95% CI, 0.871 to 0.889).

**Table 5:** **Performance of the basic life support termination-of-resuscitation rule for predicting one-month death**^**a**^

	**BLS TOR rule**	
**Validation group**	**Fulfilled 3/3 criteria**	**Did not fulfill criteria**
**(*N *** **= 105,030)**	**(*n *** **= 85,728, 81.6%)**	**(*n *** **= 19,302, 18.4%)**
Death, *n*	84,723	15,752
Survival, *n*	1,005	3,550
Sensitivity (95% CI)	0.843 (0.841 to 0.846)	
Specificity (95% CI)	0.779 (0.767 to 0.791)	
PPV (95% CI)	0.988 (0.988 to 0.989)	
NPV (95% CI)	0.184 (0.179 to 0.189)	
Area under the ROC curve (95% CI)	0.811 (0.809 to 0.813)	

^a^BLS, basic life support; CI, confidence interval; NPV, negative predictive value; PPV, positive predictive value; ROC, receiver operating characteristic; TOR, termination of resuscitation.

**Table 6:** **Performance of the basic life support termination-of-resuscitation rules for predicting one-month unfavorable neurological outcomes**^**a**^

	**BLS TOR rule**	
**Validation Group**	**Fulfilled 3/3 criteria**	**Did not fulfill criteria**
**(*N *** **= 105,030)**	**(*n *** **= 85,728, 81.6%)**	**(*n *** **= 19,302, 18.4%)**
Unfavorable (CPC categories 3 to 5), *n*	85,575	17,308
Favorable (CPC categories 1 and 2), *n*	153	1,994
Sensitivity (95% CI)	0.832 (0.829 to 0.834)	
Specificity (95% CI)	0.928 (0.917 to 0.938)	
PPV (95% CI)	0.998 (0.997 to 0.998)	
NPV (95% CI)	0.103 (0.099 to 0.108)	
Area under the ROC curve (95% CI)	0.880 (0.871 to 0.889)	

^a^BLS, basic life support; CI, confidence interval; CPC, cerebral performance category; NPV, negative predictive value; PPV, positive predictive value; ROC, receiver operating characteristic; TOR, termination of resuscitation.

## Discussion

**Figure 2:**
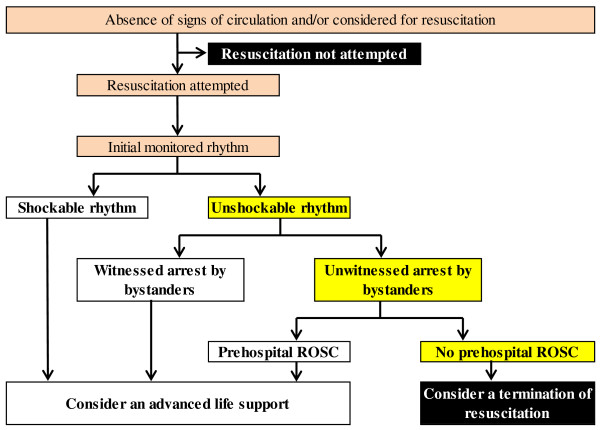
**Flowchart algorithm of new termination-of-resuscitation rule for emergency department physicians according to the Utstein template.** ROSC, return of spontaneous circulation.

We developed and validated a new rule to guide physicians in deciding whether to terminate resuscitation after OHCA immediately after patient arrival at the emergency department. A new TOR rule was defined to fulfill the following three criteria: no prehospital ROSC, unshockable initial rhythm and unwitnessed cardiac arrest by bystanders. Figure 2 shows a flowchart algorithm of how the new TOR rule should be applied. If a patient with OHCA fulfills all three criteria immediately after patient arrival at the emergency department, the physician in charge should consider terminating resuscitation before performing ALS. Our present results demonstrate that the new TOR rule has high specificity, PPV and area under the ROC curve for predicting one-month outcomes, although our TOR rule could not fully predict one-month death. We validated the BLS TOR rule using the validation data set to compare the performance of the new TOR rule. Our new TOR rule had higher specificity, PPV and area under the ROC curve than the BLS TOR rule for predicting one-month outcomes. This finding implies that the new rule is preferable to the BLS TOR rule in Japan. Unlike the international TOR rules for EMS personnel in the field, the new TOR rule presents no burden to EMS personnel for determining the futility of CPR.

TOR in the field is a crucial issue for both patients with OHCA and healthcare staff, including EMS personnel. Although TOR clinical prediction rules can minimize costs and lead to better use of EMS resources [7], ethical issues around TOR remain controversial. In 1990, an objective criterion for medical futility was defined for interventions and drug therapy imparting a less than 1% chance of survival [23], and this level remains a basis for current futility research [5]. Our newly established TOR rule showed misclassification rates of less than 1% for predicting both death (0.8% for the development group and 0.7% for the validation group) and unfavorable outcomes (0.2% for the development group and 0.1% for the validation group) at one month after OHCA.

Kajino *et al*. [19] recently investigated whether international TOR rules can predict one-month outcomes for selected OHCA patients with presumed cardiac etiologies. They showed that the BLS TOR rule [7] had a PPV of 0.990 (95% CI, 0.989 to 0.990) and a specificity of 0.878 (95% CI, 0.872 to 0.884) for one-month death. However, our new TOR rule for OHCA patients with any etiology has a slightly higher PPV and specificity than those in the Kajino *et al*. study. A possible reason for this difference is the different inclusion and exclusion criteria used between the studies. Moreover, this OHCA registry has been the source of several previous studies [18,24,25].

Both the European Resuscitation Council [26] and the AHA [5] have developed guidelines for the ethical termination of unsuccessful resuscitation to help EMS personnel identify futile resuscitation efforts in the prehospital setting. Despite international TOR guidelines, the estimated rate of adherence to the AHA guidelines at the local level is below 50% [13]. Sasson *et al*. [15] identified three distinct groups of stakeholders whose current policies may impede efforts to adopt TOR rules: payers who incentivize transport, legislators who create state mandates for transport and allow only narrow use of do-not-resuscitate orders and communities in which cultural norms are perceived to impede TOR. In a survey of emergency physicians, 92% of respondents cited fear of litigation as a reason for continuing futile resuscitation efforts in cases of cardiopulmonary arrest [14]. The prevalent “rescue culture” of EMS providers has also been a barrier to the implementation of TOR rules [15]. Furthermore, the optimal duration of CPR prior to terminating resuscitation efforts in the field has not yet been defined [27,28]. Therefore, taking these circumstances into consideration, a new TOR rule for emergency department physicians may also serve EMS personnel in the field to minimize costs and better utilize healthcare resources.

New treatments such as hypothermia [29] and extracorporeal CPR [30] for cardiac arrest, as well as improvements in prehospital system factors such as time to start CPR and time to defibrillation [26], may improve outcomes following OHCA. Therefore, our new TOR rule for physicians should be modified periodically with the emergence of new treatments and the evolution of social systems.

Although do-not-resuscitate orders and living wills are generally not used in Japan [19], end-of-life decisions are complex and can be influenced by individual; international; and local cultural, legal, traditional, religious, social and economic factors [26]. Accordingly, the new TOR rule for emergency department physicians should be validated prospectively before implementation. In addition, further discussion of end-of-life decisions and ethical considerations after futile CPR is required, including education and debriefings for healthcare professionals.

### Study limitations

The potential limitations of the current analysis are as follows. First, we did not evaluate detailed in-hospital interventions. We assumed that OHCA patients received standard ALS according to the Japanese CPR guidelines [17], which were based on the 2005 AHA guidelines [20]. Second, there may be unmeasured confounding factors that could have influenced outcomes. However, the use of uniform data collection on the basis of Utstein-style guidelines for reporting cardiac arrest, the large sample size and a population-based design were intended to minimize these potential sources of bias. Third, the relevance of our results to other communities with different emergency care protocols and different OHCA causes remains unknown. Although TOR outside the hospital is not allowed in some Asian countries [31-33], EMS systems in those countries are different from the Japanese EMS system. Therefore, studies in other countries may be required to validate our results. Fourth, emergency department physicians must pay attention to the use of the new TOR rule for patients with special conditions such as accidental hypothermia. Fifth, emergency department physicians should decide whether further medication is needed, even if patients meet the newly derived TOR rule. Finally, no measure of interrater reliability is obtainable from the data set to guide the determination of the rate of misclassification.

## Conclusions

We have developed and validated a new TOR rule consisting of three prehospital variables (no prehospital ROSC, unshockable initial rhythm and unwitnessed by bystanders) for emergency department physicians. This new TOR rule would offer a rule with a more than 99% predictor of very poor outcome. However, the implementation of this new rule in other countries or EMS systems, especially in systems with a higher OHCA survival rate than Japan’s, requires further validation studies.

## Key messages

• We analyzed prospectively recorded, nationwide, Utstein-style Japanese data over a five-year period and developed a new TOR rule, which includes three criteria (no prehospital ROSC, unshockable initial rhythm and unwitnessed by bystanders) for emergency department physicians.

• We validated the new TOR and BLS TOR rules and found that the new TOR rule showed higher specificity, PPV and area under the ROC curve for predicting one-month poor outcomes than those of the BLS TOR rule.

• This new TOR rule would offer a rule that finds a more than 99% predictor of very poor outcome.

• Further validation studies are required before this new TOR rule can be implemented in other countries or EMS systems.

## Abbreviations

AHA: American Heart Association; ALS: Advanced life support; BLS: Basic life support; CI: Confidence interval; CPC: Cerebral performance category; CPR: Cardiopulmonary resuscitation; ELST: Emergency lifesaving technician; EMS: Emergency Medical Services; FDMA: Fire and Disaster Management Agency; IQR: Interquartile range; OHCA: Out-of-hospital cardiac arrest; OR: Odds ratio; PPV: Positive predictive value; ROC: Receiver operating characteristic; ROSC: Return of spontaneous circulation; TOR: Termination of resuscitation.

## Competing interests

The authors declare that they have no competing interests.

## Authors’ contributions

YG and TM designed the study. YG, TM and YNG conducted data cleaning. YG and YNG analyzed the data. YG drafted the manuscript. YNG and TM contributed substantially to manuscript revision. YG takes responsibility for the paper as a whole. All authors approved the manuscript before submission. All authors read and approved the final manuscript.
